# How to Evaluate the Chemical Affinity of -OH and -COOH Functional Groups Toward U(VI)

**DOI:** 10.3390/molecules29235614

**Published:** 2024-11-27

**Authors:** Xuemei Cui, Xiaoying Xie, Yun Li, Yue Chen, Yan Ma, Shubin Yang

**Affiliations:** 1 School of Chemistry and Chemical Engineering, Yantai University, Yantai 264005, China; cuixuemei19991216@163.com; 2 Yantai-Jingshi Institute of Material Genome Engineering, Yantai 264006, China; cysqxr@hotmail.com; 3 Aviation Service Department, Yantai Engineering & Technology College, Yantai 264006, China; myan_april@hotmail.com

**Keywords:** -OH group, -COOH group, U(VI) enrichment, chemical affinity

## Abstract

Which functional group shows a stronger affinity for U(VI) and can be introduced into material to enhance selective enrichment? This is crucial for U(VI) capture material design and evaluation. Following these questions, we herein compared and analyzed bare graphene, graphene oxide (GO), and carboxylated graphene oxide (GO-COOH) through experimental and theoretical calculations. Experiments show that U(VI) adsorption on GO-COOH (*Q_m_* = 344.1 mg/g) mainly occurs via inner-sphere complexation with the C=O group in -COOH. The -COOH group can significantly enhance the enrichment and selectivity of U(VI), and its affinity for U(VI) is greater than that of -OH. There is a strong interaction between [UO_2_(H_2_O)_10_]^2+^ and -COOH with an interaction energy of 1.13 eV. When U(VI) is adsorbed on GO, the original C-O(H) bond in GO breaks, leading to U(VI) seizing -OH and forming a more stable complex [UO_2_(H_2_O)_10_(OH)]^1+^. However, the desorption of U(VI) from GO is easier due to the weakened interaction between [UO_2_(H_2_O)_10_(OH)]^1+^ and GO after the C-O(H) bond breakage. Briefly, the combination of experimental observations and theoretical calculations provides a comprehensive understanding of the affinity and selectivity of -COOH and -OH for U(VI), and highlights the potential of using -COOH functionalization to enhance the U(VI) enrichment and separation performance of materials.

## 1. Introduction

Radionuclide uranium is not only an important nuclear fuel resource, but also one of the main polluting elements in highly radioactive waste liquid [1]. According to the International Energy Agency, global nuclear industry capacity could expand by more than 40% by 2030, leading to rising demand for U(VI) consumption [2]. At the United Nations Climate Change Conference (COP28) in 2023, more than 20 countries including the United States signed a joint declaration to triple nuclear energy capacity by 2050. The efficient separation and recycling of U(VI) is not only conducive to the sustainable development of nuclear power, but also to the recycling of resources, environmental protection, and human health [3].

How to achieve highly selective enrichment of radioactive U(VI) in complex environmental systems and study the mechanism of material action on U(VI) from the microscopic scale is a difficult problem in the development of highly enriched materials for U(VI). According to the characteristics of target nuclides, it is of great scientific significance to select and construct highly selective enrichment materials to achieve efficient and rapid enrichment of low-concentration radionuclides in complex systems [4]. Therefore, great efforts are continuously devoted to developing several materials for uranium enrichment, such as inorganic materials [5], hyper porous materials (ca. COFs, MOFs, hyper porous carbons) [6,7], large surface area materials (graphene oxide), and magnetic nanoparticles [8]. Although extensive research studies have been carried out on materials for the high enrichment of U(VI), problems such as the selectivity of materials and their mechanism have not been well solved. Functional groups on the substance surface could affect the chemical functionality of the materials [9]. Thus, functional group modification is an efficient way to enhance the selectivity of materials by modifying special functional groups with high affinity for U(VI). Varieties of groups have been introduced for the fabrication of materials to improve their U(VI) selectivity. Chai Zhifang’s team proposed that the introduction of different amino groups into acid-resistant MOFs could significantly improve the enrichment ability and selectivity of U(VI) [10]. Sachs et al. found that the addition of a sulfhydryl functional group can improve the selectivity of the material to U(VI) due to the coordination of the sulfhydryl functional group with U(VI) [11]. The type and quantity of functional groups and their affinity to radionuclides are important factors that determine the highly selective enrichment ability of materials. However, there are currently few systematic evaluations on the functional group affinity of materials.

Hydroxyl (-OH) and carboxyl (-COOH) are the most widely used O-containing functional groups in the materials surface functionalization [12]. They are also the simple ligands that can effectively bind toward uranyl ions [13]. These functional groups are able to provide their lone pair of electrons to the empty d-orbitals of the transition metal ions, thus forming metal complexes [14]. Because the valence d-orbitals of transition metal extend well into the periphery of the atom, they can interact with ligands to form covalent bonds and a d-π* back-bond. In contrast, the 4f-orbitals of lanthanide are generally core-like, and the interaction between Ln^3+^ with ligands is largely electrostatic metal–ligand interactions and of little chemical consequence [15,16]. Although -OH and -COOH are extensively used in material functional modification for metal ions adsorption, the detailed mechanism of this phenomenon remains unclear, especially for the actinide and lanthanide metal ions with f-orbitals. This is an important drawback for improving the adsorption properties of these materials.

**Figure 1:**
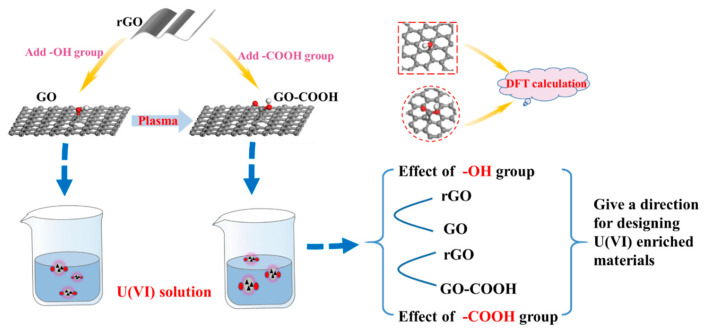
Schematic illustration of this research approach.

Compared with -OH and -COOH, which functional group has a stronger affinity for U(VI), and which functional group can be modified to improve the selective enrichment of U(VI)? This is of great significance for pollution control and recycling of U(VI). They are also the main questions we followed in the current research. In order to solve these questions, we here prepare graphene oxide (GO) and carboxylated graphene oxide (GO-COOH). GO is an important derivative of graphene with rich O-containing functional groups, mainly epoxide and hydroxy groups on the basal plane, with a small number of carboxyl moieties at the edges [17]. Previous studies have found that the enrichment of heavy metal ions by GO is mainly through the -OH functional group on the GO surface, while the role of the epoxy functional group is relatively small [8,18]. Therefore, we select -OH and -COOH as representative functional groups of graphene to simulate GO and GO-COOH, respectively. The adsorption activity is evaluated with bare graphene as a reference. The effect of -OH and -COOH functional groups on U(VI) enrichment is analyzed and the affinity sequence between the two functional groups with U(VI) is given. To clarify the enrichment mechanisms of U(VI) with -OH and -COOH groups, the chemical species and microstructures of U(VI) on the different O-containing functional groups at the molecular level are studied in depth and detail by DFT calculations. Our approach is depicted in Figure 1. Different environmental factors (e.g., temperature, pH, competitive ions, four different water systems), adsorption thermodynamics, adsorption kinetics, and recyclability of the materials are also studied and discussed in detail to understand the mechanism of U(VI) sorption. Research is very important for the design and evaluation of U(VI) capture materials, and there is also an urgent need for the advancement of high-level waste disposal technology.

## 2. Experimental Sections

### 2.1. Synthesis of Graphene Oxide (GO) and Simple Thermal Reduced GO (rGO)

The highly-oxidized GO is synthesized via a modified Hummers method [19]. Briefly, expanded graphite and NaNO_3_ are added to a concentrated H_2_SO_4_ ice-water bath, and then KMnO_4_ is slowly added under constant mechanical stirring for 24 h. The mixture is then diluted with Milli-Q water and heated up to 98 °C with stirring for an additional 4 h. Finally, H_2_O_2_ solution is added to remove excess MnO_4_^−^ anions, and the highly oxidized GO is collected by centrifuging, repeatedly washing with Milli-Q water and finally dried in an oven at 60 °C for 24 h. Subsequently, the bare graphene is prepared by a simple thermal reduction method. The as-prepared GO is heated from room temperature to 300 °C in an oven, held for 1 h, and finally, the reduced graphene oxide (rGO) is collected. The functional groups are removed by high-temperature evaporation, resulting in tough graphene nanosheets with a layered porous structure.

### 2.2. Synthesis of Carboxylated Graphene Oxide (GO-COOH)

The carboxylated graphene oxide (GO-COOH) is synthesized using a plasma modification method. Typically, GO powder is transferred into a customized plasma reactor. A high-voltage pulsed DC voltage of 100 W is applied to the plasma coil through Ar, until the vacuum degree is maintained at 25 Pa, and the material is activated for 10 min. The surface of the GO is activated by Ar ion bombardment to enhance its reactivity. The plasma-treated GO is then quickly transferred into a carboxylated chitosan solution and stirred at 60 °C for 12 h. Finally, it is washed with distilled water, centrifuged, and dried in a vacuum oven at 60 °C to obtain GO-COOH.

## 3. Computational Details

The structure optimizations and electronic-structure calculations are performed using the Vienna Ab-initio Simulation Package (VASP5.4). The projector augmented-wave (PAW) pseudo potentials are used to tackle the electron–ion interaction, and the electron exchange and correlation are handled by the Perdew–Burke–Ernzerhof (PBE) functional of the generalized gradient approximation (GGA) [20]. The cutoff energy is set to 400 eV. A 1 × 1 × 1 k-point mesh is used for all simulations. The geometries are fully optimized until the forces on each atom are less than 0.02 eV/Å. In addition, the calculation takes into account the van der Waals interaction [21].

**Figure 2:**
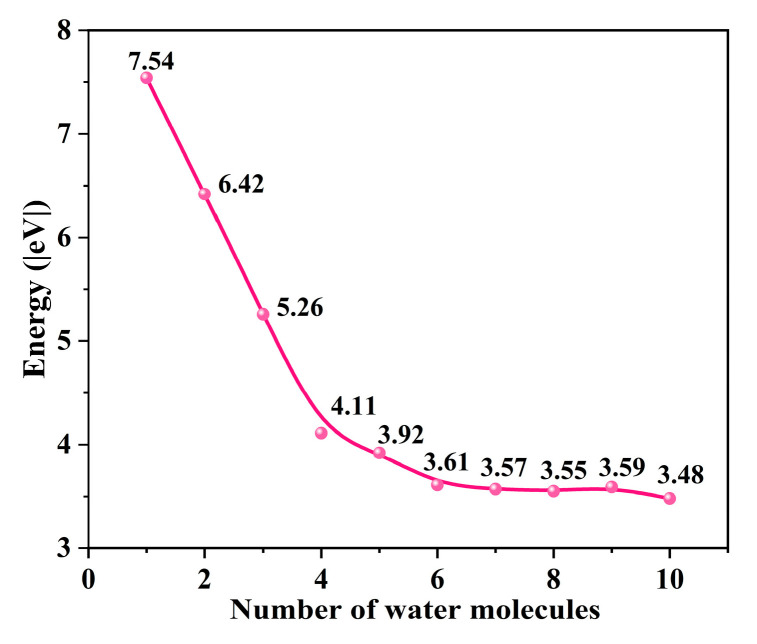
The energy change as the number of H_2_O increases.

To determine the optimal structure of the uranyl ion, we gradually added explicit water molecules to its structure and calculated the hydration energy change. While the number of water molecules is larger than 5, the hydration energy changes are around 3.5 eV (Figure 2), indicating that the hydration environment becomes stable enough. Finally, the uranyl system with ten explicit water molecules [UO_2_(H_2_O)_10_]^2+^ is selected to be the uranyl model for further calculation. In this work, we choose -OH and -COOH groups as representative groups of graphene to simulate GO and GO-COOH. A 7 × 7 graphene supercell is constructed as the adsorption substrate, and the lattice parameters (a = b) of GO and GO-COOH are 17.22, and 17.22 Å, respectively. In addition, in order to avoid mutual influence between layers, the thickness of the vacuum layer in the vertical direction (c direction) is set to 30 Å. The adsorption energy (*E_ads_*) is calculated as follows in Equations (1) and (2):

(1) $$E_{ads}=E_{GO+UO_{2}^{2+}}-E_{GO}-E_{UO_{2}^{2+}}$$

(2) $$E_{ads}^{\prime }=E_{C}-E_{0}$$

where *E_GO_*, $E_{UO_{2}^{2+}}$, and $E_{GO+UO_{2}^{2+}}$ are the energies of GO, U(VI) ions, and the complex of GO with U(VI), respectively. $E_{ads}^{\prime }$, *E_C_*, and *E*_0_ are the energies of the total adsorption energy of the system, coordination adsorption energy, and the zero-point adsorption energy, respectively.

## 4. Results and Discussion

### 4.1. Characterization

**Figure 3:**
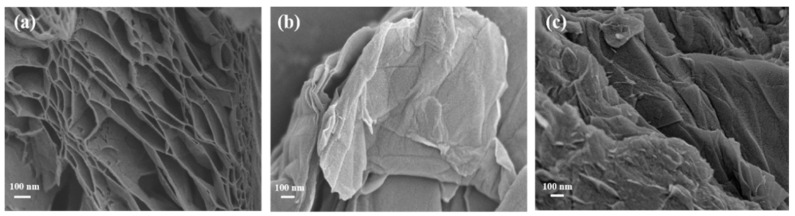
SEM images of rGO (**a**), GO (**b**), and GO-COOH (**c**).

The bare-graphene is prepared by high-temperature evaporation to remove functional groups and enable the formation of tough graphene nanosheets with hierarchically porous structures (Figure 3a). Obviously, rGO shows a 3D hierarchical porous graphene structure [22]. The range of pore size is 25–700 nm, and the pore walls consist of thin layers of graphene sheets. Completely different, GO’s surface is much smoother, and the honeycomb structure is gone. GO shows a large number of slightly wrinkled and distinguishable nanolayer structures (Figure 3b). Due to the interaction of hydrophobic regions between the GO sheets, the flexibility of the GO sheets can produce some wrinkles on the surface [14]. Compared with GO, GO-COOH has a rougher surface and more irregular folds (Figure 3c), which might be a result of the many -COOH groups introduced during the oxidation process [23]. These adsorption sites provided by -COOH groups, exposed on the material surface, may improve the adsorption morphology and distribution of U(VI) on the composite material surface, thereby enhancing the overall adsorption performance of the material.

**Figure 4:**
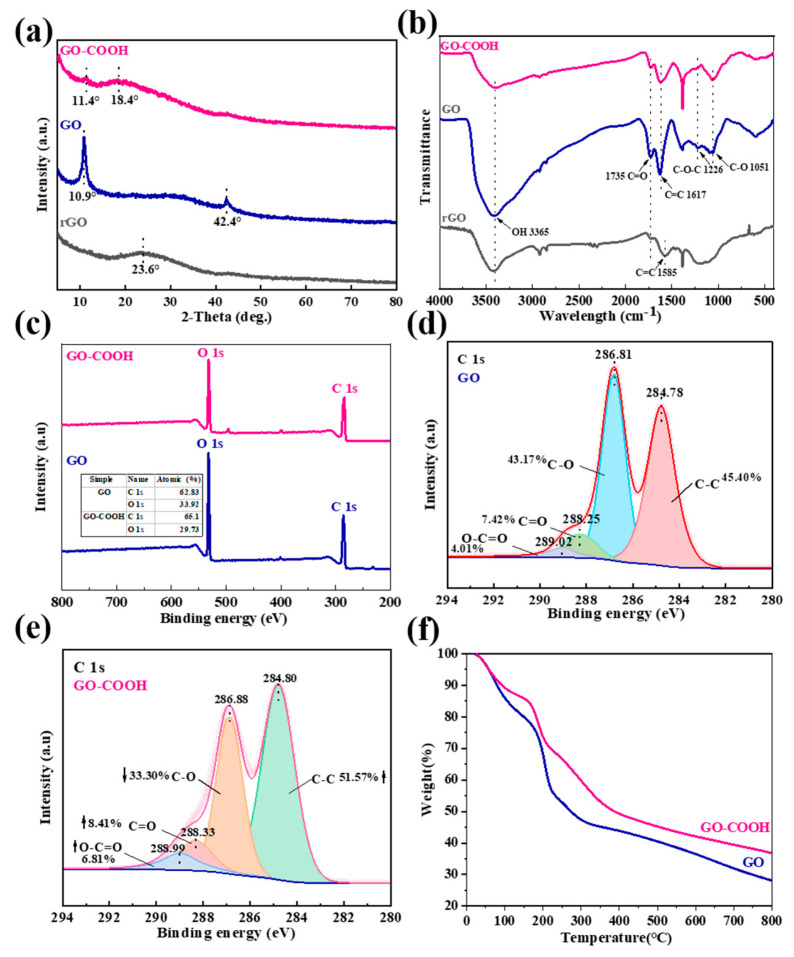
XRD patterns (**a**), FT-IR spectra (**b**), Wide-scan XPS spectra (**c**), XPS C 1s of GO (**d**), XPS C 1s of GO-COOH (**e**), TGA curves (**f**).

The microstructure changes in the GO flakes are again revealed by XRD analysis, as shown in Figure 4a. GO has a strong peak at 2θ = 10.8°, which corresponds to the (002) plane of GO [24]. This “GO” peak disappears in rGO and a new peak develops at a larger 2θ = 23.6°, which indicates the reduction of GO, and the creation of extended graphene sheets. Notably, the XRD pattern only shows a broad “graphene” peak, which suggests that reduction of the 3D porous rGO films has occurred. After the plasma modification, a very weak “GO” peak and a broad peak at 2θ = 18.4° are observed in GO-COOH [25]. The broad and diffuse peak dominants denote the changes in the GO structure and the completion of GO modification. These results are also supported by FT-IR and XPS analysis. In the FT-IR spectrum of rGO, the stretching vibration of the C=C bond (1585 cm^−1^) is attributed to the in-plane vibration of sp^2^ hybridized carbon atoms in the graphene structure (Figure 4b) [26]. GO exhibits a stronger stretching vibration of the C=C bond (1617 cm^−1^). The introduction of C=O into a molecular structure can significantly alter the spatial environment, electron distribution, and vibration modes surrounding the C=C bond [27]. GO-COOH shows a weaker -OH peak (3365 cm^−1^) and a nearly disappeared C-O-C peak (1226 cm^−1^) in the FT-IR spectrum, indicating the conversion of some -OH groups to -COOH in the structure [9]. These results reflect the differences in chemical structure between rGO, GO, and GO-COOH, especially the presence and transformation of functional groups. Further evidence is provided by the significant changes observed in the C1s and O1s content between GO and GO-COOH (Figure 4c). Notably, there are also substantial differences in the XPS C1s peak composition corresponding to oxygen-containing groups (Figure 4d). The peak at 286.81 eV comes from the C-O bond, related to the oxygen-containing functional groups in GO that provide suitable anchoring sites for function [28]. For GO-COOH, the intensity of the C-C peak (284.80 eV) increases while the C-O (286.88 eV) signal weakens (Figure 4e). This is due to the reduction in epoxide deoxidation during plasma treatment, which leads to a decrease in C-O content, accompanied by an increase in the average fraction of the sp^2^ domain. The C=O signal increases at 288.33 eV and O-C=O appears at 288.99 eV, which proves the formation of additional carboxylate moieties [29]; thus, the carboxyl functional groups were successfully modified by plasma technology.

TGA analysis of the material is performed to assess its thermal stability. It can be seen that with the increase in temperature, the weightlessness of GO is manifested in three different stages (Figure 4f). The first stage is from room temperature to about 100 °C, during which the weight loss can be attributed to the evaporation of water vapor, and the second stage, from 150 °C to 280 °C, is due to the loss of oxygen-containing functional groups [30]. Finally, the third decomposition stage observed is from 400 to 790 °C, and its loss is related to the combustion of GO’s carbon skeleton. Therefore, the thermal reduction of GO, carried out at 300 °C for 1 h, can eventually produce a highly layered porous graphene network (Figure 3a and Figure 4f). GO-COOH has a similar thermal decomposition process to GO, except more pronounced losses are observed from 150 to 280 °C, implying the presence of richer oxygen-containing functional groups, such as -COOH in composites. These findings are consistent with literature reports [31].

### 4.2. Batch Adsorption Studies

**Figure 5:**
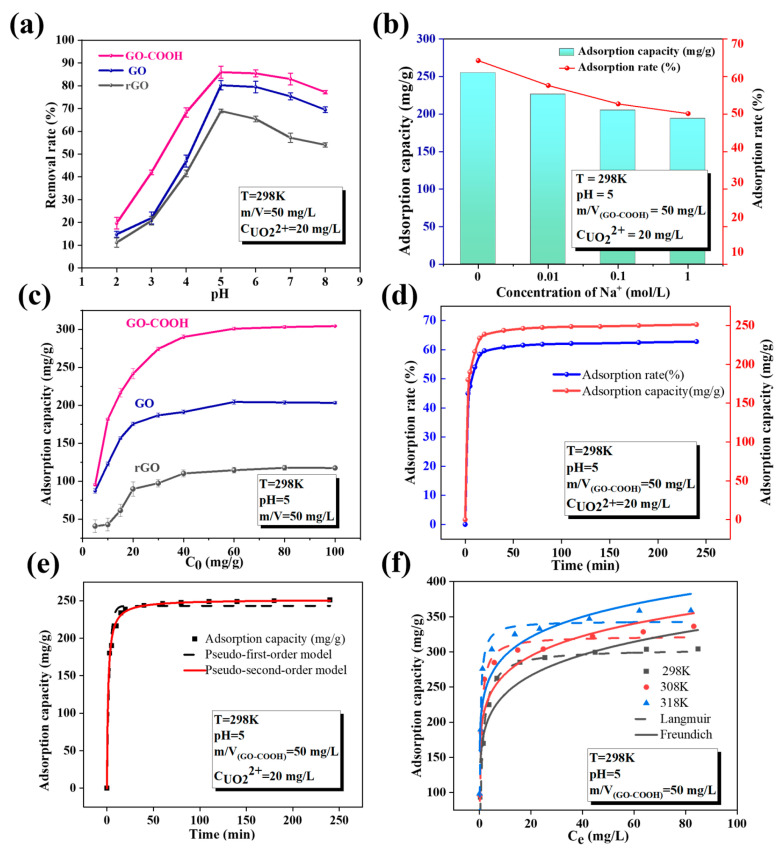
Effect of pH (**a**), ion strength (**b**), initial concentration (**c**), contact time (**d**), Kinetic models simulated plots (**e**), Adsorption isotherms (**f**).

In order to evaluate the affinity of -OH and -COOH to U(VI), batch experiments are used to study the sorption behavior of rGO, GO, and GO-COOH for U(VI). Different environmental factors such as pH, temperature, competitive ions, and four different water systems are studied. As shown in Figure 5a, the pH value plays a crucial role in regulating the adsorption of U(VI) by GO-based materials, indicating that the adsorption process of U(VI) on GO is mainly controlled by surface complexation [27]. Furthermore, the adsorption efficiency reaches its peak at a specific pH range, making it crucial to control the pH during the adsorption process. The optimal enrichment efficiency occurs around pH 5.0. The solution pH has a great influence on the surface charge of materials and the presence of nuclide ions in aqueous solutions [32]. Adsorption processes that are significantly influenced by pH generally follow an adsorption mechanism dominated by surface complexation [27]. Literature reports indicate that when the pH value is less than 5.0, UO_2_^2+^ is the dominant species and competes with H^+^ in the solution for adsorption sites [33]. As the pH value increases, more U(VI) is converted into hydrolyzed complexes and polynuclear hydroxide complexes, such as UO_2_(OH)^+^ and UO_2_(OH)_2_, leading to a significant reduction in the amount of U(VI) that can be adsorbed. This indicates that the pH value of the solution not only affects the presence of U(VI) but also directly influences the ease of its adsorption.

Under the same pH conditions, the adsorption performance of the three materials for U(VI) exhibits a significant difference (Figure 5a,c), with the specific order being: GO-COOH (303.93 mg/g) > GO (201.54 mg/g) > rGO (119.12 mg/g). This indicates that the introduction of -COOH is superior to -OH, significantly enhancing the adsorption performance of U(VI). These two functional groups are able to donate their lone pair electrons to the 4f orbital of U(VI), forming electrostatic complexes through electrostatic complexation [14]. This mechanism is distinct from that of oxygen-containing functional groups forming complexes with transition metals via covalent bonds or d-π* bonds [34]. This interaction is significant for enhancing the adsorption capacity and selectivity of materials toward U(VI), as it establishes a strong connection between the functional groups and U(VI) ions. This connection aids the material in more effectively adsorbing and selectively removing U(VI), thereby offering potential applications in fields such as environmental protection and nuclear waste management.

The concentration of background electrolyte has a significant impact on U(VI) enrichment by GO-COOH (Figure 5b), as it directly affects the ionic strength. Ionic strength is a crucial factor affecting the adsorption behavior of the material, as it can alter the thickness of the double layer and the interfacial potential of the adsorbent, thus impacting the binding between the adsorbate and the adsorbent [35]. Therefore, it can be inferred that the adsorption of U(VI) by GO-COOH occurs through outer-sphere complexation. Moreover, this adsorption process by GO-COOH is very fast, reaching adsorption equilibrium within 30 min (Figure 5d) and the adsorption curve is more consistent with the pseudo-second-order kinetic model (Figure 5e and Table 1), indicating that the adsorption kinetics are mainly controlled by chemical action, and it can also be inferred that the adsorption process involves surface complexation reactions between U(VI) and GO-COOH [36]. In addition, an increase in temperature helps to improve the adsorption performance (Figure 5f and Table 2), and when combined with the thermodynamic parameters (ΔH, ΔS, and ΔG) obtained from the slope and intercept of the lnK_d_ vs. 1/T curve (Table 3), it proves an endothermic adsorption process [37]. The maximum adsorption capacity (Q_max_) of GO-COOH is 344.1 mg/g (Table 4), which is significantly better than that of various materials.

**Figure 6:**
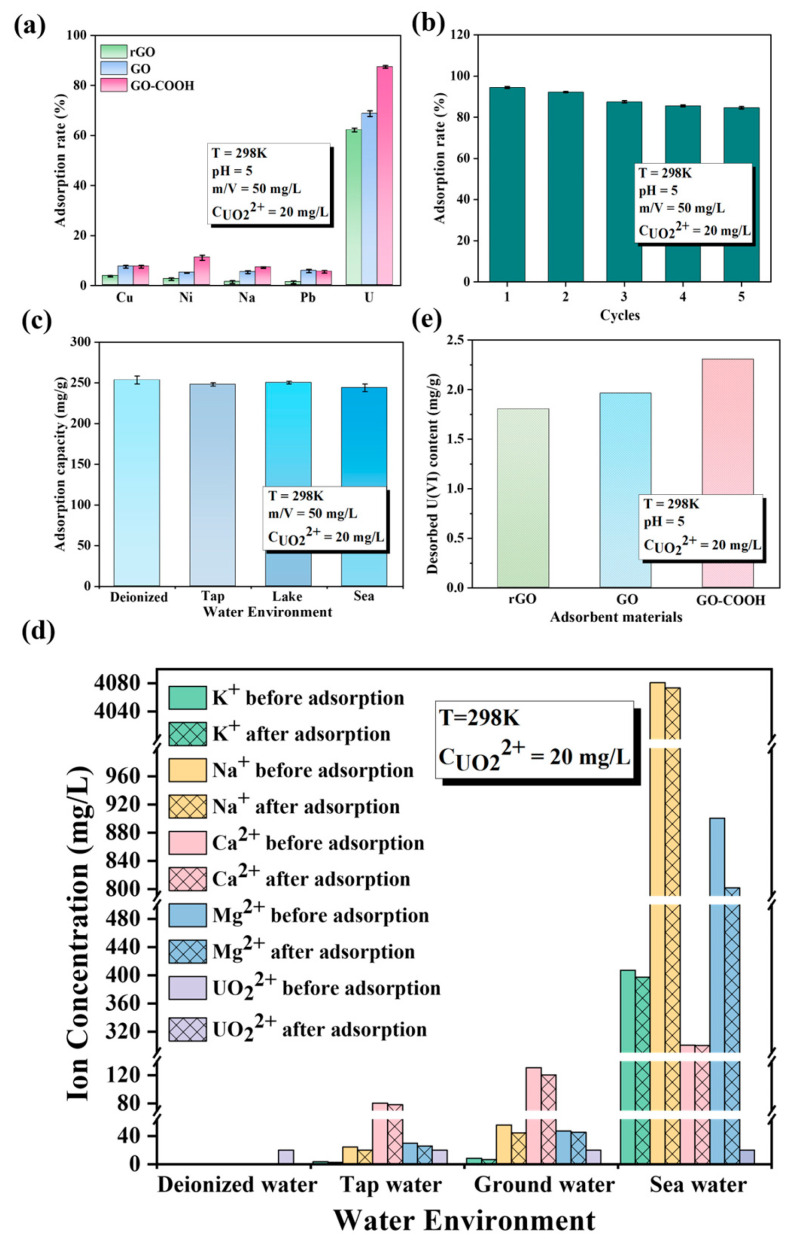
Effect of competitive cations (**a**), different water systems (**b**), recyclable times (**c**), and water systems in U(VI) sorption by GO-COOH (**e**). Desorption Performance Comparison (**d**).

GO-COOH not only exhibits outstanding enrichment performance for U(VI) but also demonstrates excellent selectivity, stability, and recovery performance (Figure 6). Even in various complex aqueous systems, including deionized water, tap water, underground water from Yantai Sanyuan Lake, and seawater from the Bohai Sea in China, it displays remarkable adsorption efficiency (Figure 6c). Notably, in these four different types of water, GO-COOH maintains consistent adsorption capacity for U(VI). This finding not only emphasizes that the ionic strength of the solution has a minor impact on U(VI) adsorption but also suggests the versatility and practicality of GO-COOH in water treatment applications. Before and after adsorption, the concentrations of K^+^, Na^+^, Ca^2+^, and Mg^2+^ in the four aqueous systems remain largely unchanged, while the concentration of U(VI) decreases significantly (Figure 6d). This once again verifies the exceptional selectivity of GO-COOH for U(VI). Furthermore, after adsorption, it was observed that U(VI) is harder to remove from GO-COOH than from GO (Figure 6e), indicating that the -COOH group has a stronger affinity for U(VI) than the -OH group. This excellent adsorption performance of GO-COOH may be attributed to the interaction between the -COOH group and U(VI), making it a promising material for water treatment under various environmental conditions. The affinity and selective enrichment mechanism of the -COOH functional group with U(VI) are subsequently analyzed and elaborated through microstructural characterization and DFT calculations.

### 4.3. Adsorption Mechanism

**Figure 7:**
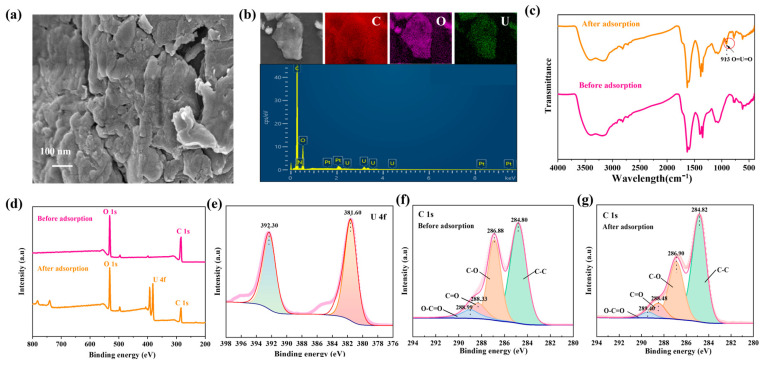
SEM image (**a**), EDS and mapping (**b**), FT-IR patterns (**c**), Wide-scan XPS spectra of GO-COOH (**d**), XPS U4f (**e**), XPS C1s (**f**,**g**), before and after U(VI) adsorption on GO-COOH.

To understand the adsorption process of U(VI) on GO-COOH, we conducted detailed SEM, FT-IR, and XPS analyses before and after adsorption. These analyses help us comprehensively understand the adsorption behavior of U(VI) on GO-COOH and the underlying chemical mechanisms. After adsorption, significant changes occurred in the microstructure of GO-COOH, with more pronounced aggregation on the material surface (Figure 7a). The distribution of adsorbed U(VI) was consistent with that of O on the material surface (Figure 7b), indicating that the adsorption of U(VI) on the material originated from the oxygen-containing functional groups on its surface. Additionally, a new peak emerged in the FT-IR spectrum at 913 cm^−1^ (Figure 7c), which corresponds to the stretching frequency of the [O=U=O]^2+^ linear structure [45]. Its presence confirms the incorporation of uranyl ions into the composite material. In the XPS spectrum, a set of U(VI) spin-orbit splitting peaks, namely, the U4f_7/2_ (381.60 eV) and U4f_5/2_ (392.30 eV) peaks, are observed (Figure 7d,e), and no U(IV)-related peaks are detected [46]. This indicates that U(VI) ions are bound to the oxygen-containing groups on the material, and no chemical reduction of uranium occurs during the adsorption process. Furthermore, in the XPS C1s spectrum, it is observed that after U(VI) sorption, the peak positions of C=O and O-C=O shift significantly toward higher binding energies, while the peak positions of C-O and C-C remain relatively stable. This phenomenon provides important information about the adsorption behavior of U(VI) on GO-COOH, indicating changes in the chemical bonding state or chemical environment. The positive shift in the C=O and O-C=O peaks confirms their complexation with U(VI).

**Figure 8:**
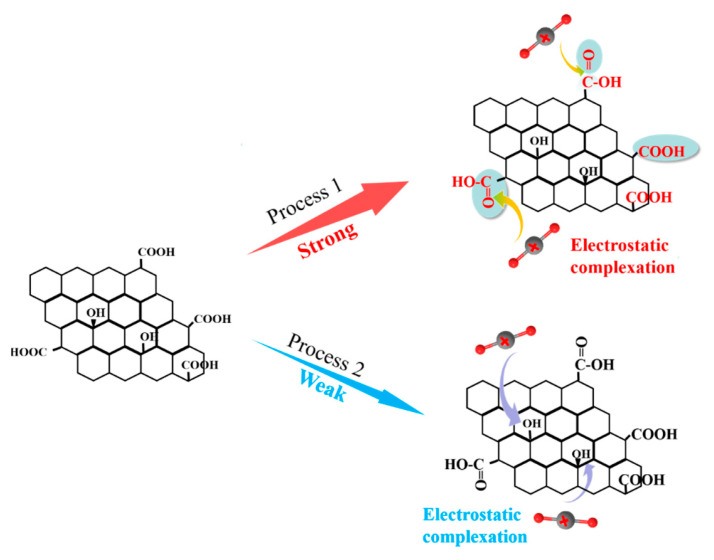
Schematic diagram for the U(VI) adsorption mechanism by GO-COOH.

In summary, the -COOH groups play a crucial role in the immobilization of U(VI), and U(VI) ions are primarily adsorbed by GO-COOH through complexation with the C=O group in -COOH, resulting in the formation of more stable complexes (Figure 8). The -COOH group can donate its lone pair of electrons to the 4f orbital of U(VI), forming an electrostatic complex through electrostatic complexation [14]. These results suggest that U(VI) should have a strong affinity and selectivity for the -COOH functional groups, which is consistent with previous adsorption experiment results.

### 4.4. DFT Calculation

**Figure 9:**
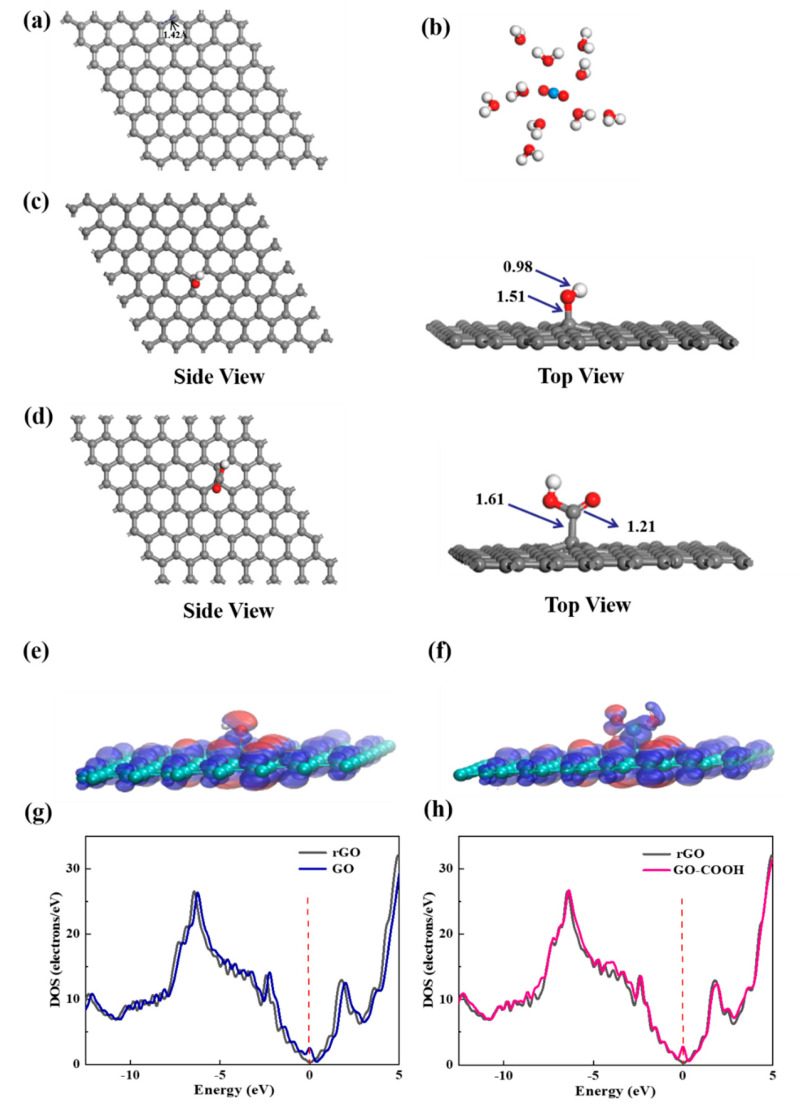
The most stable structures of graphene (**a**), [UO_2_(H_2_O)_10_]^2+^ (**b**), GO (**c**), and GO-COOH (**d**); the ESP distribution (**e**,**f**) and the DOS schematic diagrams (**g**,**h**) for GO and GO-COOH.

To elucidate the selective enrichment mechanisms of U(VI) with -OH and -COOH groups, the chemical species of U(VI) on bare graphene, GO, and GO-COOH at the molecular level are studied by DFT calculations. The initial stable structures involved in the calculation are shown in Figure 9. Graphene exhibits a standard hexagonal network structure with a C-C bond length of 1.42 Å. The molecular electrostatic potential (ESP) diagram of GO and GO-COOH reveals that the -OH and -COOH groups exhibit strong electrophilic ability and high chemical reactivity (Figure 9e,f). Nucleophilic regions are depicted in blue, indicating stronger repulsive forces, while electrophilic regions are shown in red, signifying greater attraction in these areas. Further density of state (DOS) analysis reveals that the DOS spectra of GO and GO-COOH exhibit similarities to that of rGO, indicating certain electronic structural similarities (Figure 9g,h). This is due to their common graphene skeleton structure. The introduction of -OH or -COOH groups is observed to shift the conduction band to the right, suggesting that the addition of functional groups enhances the conductivity of graphene to a certain extent.

**Figure 10:**
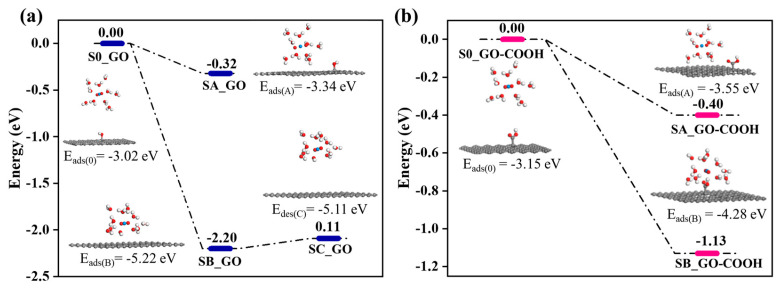
The energy change of GO (**a**) and GO-COOH (**b**)-adsorbed UO_2_^2+^.

**Figure 11:**
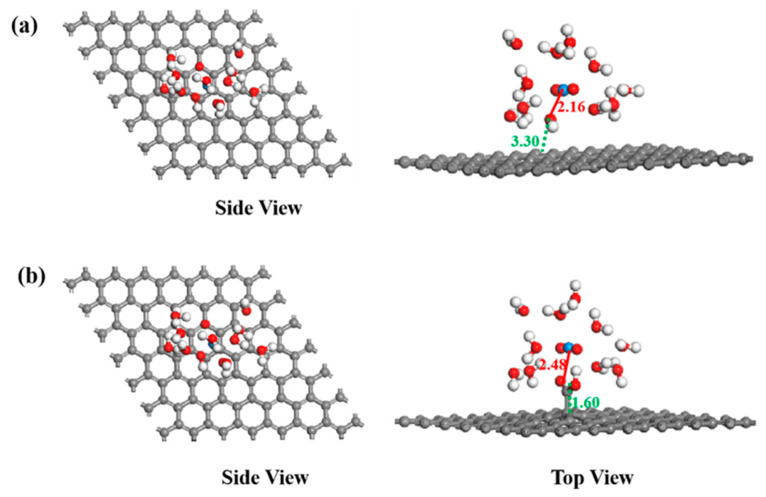
The structures of GO (**a**) and GO-COOH (**b**) after U(VI) sorption.

**Figure 12:**
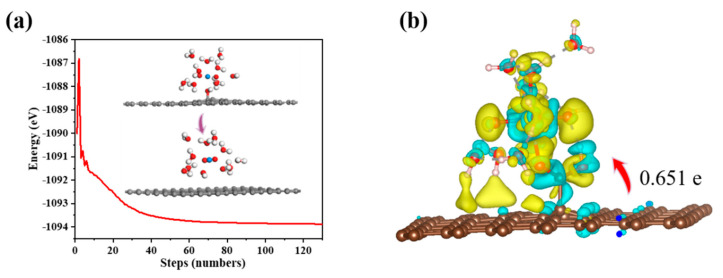
(**a**) Energy of the optimization process for the adsorption of UO_2_^2+^ on GO; (**b**) Charge density difference for the adsorption of UO_2_^2+^ on GO-COOH.

**Figure 13:**
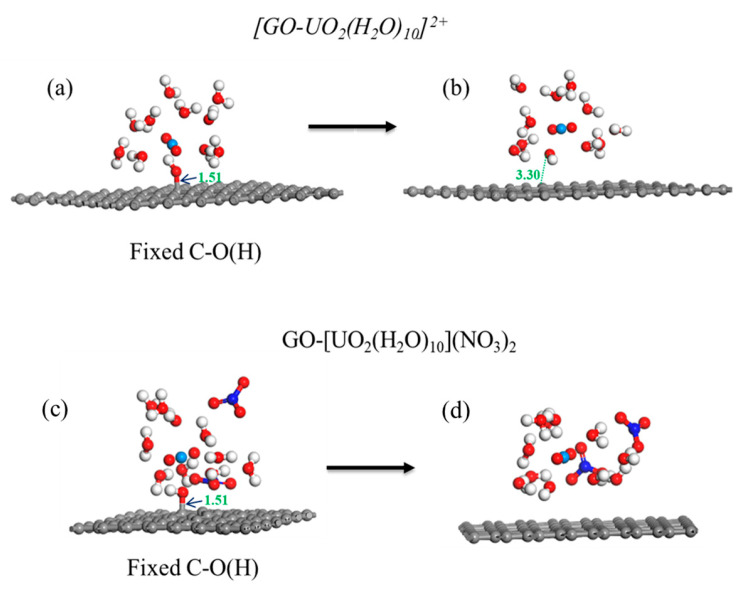
Geometry optimization with/without C-O(H) constraints for [GO-UO_2_(H_2_O)_10_]^2+^ (**a**,**b**) and GO-[UO_2_(H_2_O)_10_](NO_3_)_2_ (**c**,**d**).

In the calculation, when the [UO_2_(H_2_O)_10_]^2+^ ions move from an infinite distance to a position 10 Å away from the Z-axis of the composite material, its interaction energy is calculated to be 3.02 eV (Figure 10a), and this state (S0_GO) is taken as the zero-point to evaluate the adsorption energy of [UO_2_(H_2_O)_10_]^2+^ on GO. While [UO_2_(H_2_O)_10_]^2+^ interacts with the graphene plane but is far from the OH group (SA_GO state in Figure 10a), the interaction energy is about 0.32 eV, indicating a weak interaction. When the [UO_2_(H_2_O)_10_]^2+^ approaches the OH group, a hydroxyl abstracting reaction occurs. In the optimized adduct of [UO_2_(H_2_O)_10_]^2+^ and GO (SB_GO state in Figure 10a), the distance between the U atom and hydroxyl O atom is 2.16 Å, but the distance between the hydroxyl O atom and carbon atom of GO is 3.30 Å (Figure 11), indicating that the hydroxyl O atom coordinates with the U atom, and original C-O(H) bond in GO is broken. The SB_GO state is more stable than S0_GO by 2.20 eV. The hydroxyl abstracting reaction is likely to be a barrierless reaction since the C-O(H) bond is broken in the geometry optimization (Figure 12a). To further confirm this barrierless process, we optimized the [GO-UO_2_(H_2_O)_10_]^2+^ complex with a constraint C-O(H) distance of 1.51 Å (Figure 13), and then performed further optimization by removing the C-O(H) constraint. The finally optimized structure indicated that the C-O(H) bond breaks. In addition, we repeated the above constraint geometry optimization by adding counter ion NO_3_^−^ into the system (GO-[UO_2_(H_2_O)_10_](NO_3_)_2_). Consistently, the C-O(H) bond also breaks when the counter ions are included in the system. The interaction energy of the graphene plane far from the COOH group on graphene (SA_GO-COOH) is 0.40 eV (Figure 10b), similar to that of SA_GO. Differently, in the adduct of [UO_2_(H_2_O)_10_]^2+^ and COOH on GO-COOH (SB_GO-COOH), the distance between the U atom and O(=C) is 2.48 Å, but the –COOH group still bonds well with graphene (Figure 11b), and the interaction is 1.13 eV (Figure 10b).

Charge density difference and Bader charge analysis reveal that the adsorption of U(VI) leads to charge accumulation and depletion, primarily occurring near the new -COOH groups (Figure 12b). Approximately 0.651 e electrons are transferred from the composite material to the U(VI) ion, indicating a strong interaction between the adsorbed U(VI) ion and GO-COOH. In the charge density difference analysis, yellow and blue regions represent electron-rich and electron-deficient areas, respectively.

Furthermore, after adsorption, the desorption of U(VI) from rGO is easier than that from GO and GO-COOH (Figure 6d), which is consistent with the weaker interaction energies of SA_GO and SA_GO-COOH compared with SB_GO and SB_GO-COOH. Even though SB_GO is more stable than SB_GO-COOH, the desorption of U(VI) from GO is easier than that from GO-COOH. This is because the broken C-O(H) bond weakens the interaction of [UO_2_(H_2_O)_10_(OH)]^1+^ moiety with GO, as indicated by the small energy increase for separating [UO_2_(H_2_O)_10_(OH)]^1+^ moiety from graphene plane (SC_GO state) in Figure 10. Therefore, the desorption of U(VI) from GO-COOH is the most difficult. These phenomena demonstrate that, compared to -OH groups, -COOH groups have a higher chemical affinity for U(VI).

## 5. Materials and Methods

### 5.1. Materials

Graphite was purchased from Shanghai Shanpu Chemical Co., Ltd. (Shanghai, China). Chemicals such as sodium nitrate, potassium permanganate, concentrated sulfuric acid, nitric acid, hydrogen peroxide, uranium nitrate hexahydrate (UO_2_(NO_3_)_2_·6H_2_O), and carboxylated chitosan were purchased from Sinopharm Chemical Reagent Co., Ltd. (Shanghai, China). All chemicals were used directly without further purification.

### 5.2. Adsorption Study

Batch experiments of U(VI) adsorption were performed in polyethylene test tubes. The adsorbent powder was immersed into UO_2_(NO_3_)_2_.6H_2_O solution in a test tube to achieve the desired concentration of different components, and the initial pH value of the solution was adjusted with negligible NaOH or HNO_3_. After 24 h of oscillation, the suspension was centrifuged. The final concentration of U(VI) ions was determined by an ultraviolet spectrophotometer at 650 nm (Lambda 365, PerkinElmer, Waltham, MA, USA).

The adsorption capacity *Q_e_* (mg/g) and the removal percentage *R* (%) of U(VI) can be expressed as the following equations:

(3) $$Q_{e}=\left(C_{0}-C_{e}\right).\frac{V}{m}$$

(4) $$R=\frac{C_{0}-C_{e}}{C_{0}}\times 100\%$$

The desorption ratio (*DR*) calculation equation is given as Equation (5):

(5) $$DR=\frac{Q_{e}-Q_{e}^{\prime }}{Q_{e}}$$

where *C*_0_ and *C_e_* are the initial and equilibrium concentrations (mg/L) of U(VI), respectively, in the aqueous phase; V and m represent the volume (L) of the solution and the weight (mg) of adsorbents. $Q_{e}^{\prime }$ (mg/g) is the adsorption capacity when the desorption equilibrium is reached. Experiments are performed in duplicates.

The pseudo-first-order kinetic model (Equation (6)) and a pseudo-second-order kinetic model (Equation (7)) with nonlinear regression analysis are expressed as:

(6) $$ln\left(Q_{e}-Q_{t}\right)=lnQ_{e}-\frac{k_{1}t}{2.303}$$

(7) $$\frac{t}{\;Q_{t}}=\frac{1}{k_{2}Q_{e}^{2}}+\frac{t}{Q_{e}}$$

where *Q_t_* and *Q_e_* are the adsorption amounts of U(VI) (mg/g) at time *t* and equilibrium, respectively; *k*_1_ (min^−1^) and *k*_2_ (g/(mg·min)) represent the rate constants for the pseudo-first-order and pseudo-second-order models, respectively.

The Langmuir and Freundlich isotherm models are expressed as:

(8) $$Q_{e}=\frac{K_{L}Q_{m}C_{e}}{1+K_{L}C_{e}}$$

(9) $$Q_{e}=K_{F}C_{e}^{1/n}$$

where *Q_m_* represents the maximum adsorption capacity of the adsorbent (mg/g) by Langmuir isotherm model, *K_L_* (L/mmol) and 1/*n* are the isotherm parameters, *K_F_* (mmol/g) is the adsorption capacity by Freundlich isotherm model.

To evaluate the thermodynamic feasibility and the nature of the sorption process, several thermodynamic parameters, such as the Gibbs free energy (∆*G*), the standard enthalpy (∆*H*) and entropy change (∆*S*) are calculated by the following equations:

(10) $$K_{d}=\frac{C_{0}-C_{e}}{C_{e}}\times \frac{V}{M}$$

(11) $$lnK_{d}=\frac{∆S}{R}-\frac{∆H}{RT}$$

(12) $$∆G^{{}^{\circ}}=∆H^{{}^{\circ}}-T{∆S}^{{}^{\circ}}$$

where *K_d_* is the distribution coefficient (L/g), *R* is the gas constant in kJ/(mol⋅K) and *T* is the temperature (K).

## 6. Conclusions

A detailed analysis of the affinity difference between -OH and -COOH for U(VI) was conducted through experiments and theoretical calculations. The results show that the -COOH functional group significantly improves the enrichment performance and selectivity of U(VI), and the affinity of -COOH for U(VI) is superior to that of –OH. [UO_2_(H_2_O)_10_]^2+^ and -COOH interact strongly (1.13 eV), approximately 0.651 e transfer from GO-COOH to U(VI), with U(VI) adsorbed by GO-COOH via inner-sphere complexation with the C=O group in -COOH. Moreover, the adsorbed U(VI) is difficult to be desorbed from the product ([GO-COOH···UO_2_(H_2_O)_10_]^2+^). In comparison, the adsorption of U(VI) on GO involves an OH-abstraction process, making U(VI) in the product more easily desorbable. Thus, -OH groups exhibit a relatively weaker affinity for U(VI). In summary, the type and affinity of functional groups for radioactive nuclides are crucial for high enrichment capability. An effective strategy in designing U(VI) enriched materials is to introduce -COOH groups. This research offers new ideas and directions for their design and evaluation.

## Figures and Tables

**Table 1:** Kinetic parameters for the adsorption of U(VI) onto GO-COOH.

Adsorbents	Pseudo-First Order			Pseudo-Second Order		
	R_1_^2^	k_1_	Q_1e_	R_2_^2^	k_2_	Q_2e_
GO-COOH	0.978	0.368	243.21	0.997	0.0029	251.77

**Table 2:** Thermodynamic model parameters of U(VI) adsorption onto GO-COOH.

T(K)	Langmuir			Freundlich		
	R^2^	K_L_ (L/mg)	Q_m_	R^2^	K_F_ (mg/g)	n
298	0.981	0.941	304.133	0.857	169.795	6.646
308	0.984	1.916	322.734	0.835	198.304	7.588
318	0.954	3.107	344.130	0.862	221.669	8.076

**Table 3:** Thermodynamic parameters of U(VI) adsorption by GO-COOH.

T(K)	K_d_	ΔG^0^ (kJ·mol^−1^)	ΔH^0^ (kJ·mol^−1^)	ΔS^0^ (J·mol^−1^K^−1^)
298	26.045	−8.141		
308	34.693	−8.940	15.672	79.91
318	38.702	−9.739		

**Table 4:** The maximum adsorption capacity (Q_max_) of U(VI) on various adsorbents.

Adsorbent	Adsorption Conditions			Q_max_	References
	pH	T (K)	m/v (mg/L)	(mg/g)	
Fe_3_O_4_/graphene oxide	5	293	50	69.5	[38]
Amidoximated magnetite/GO	5	298	200	284.9	[39]
HO-CB(6)/GO	5	298	100	301.6	[40]
PAM/GO composites	5	295	200	166.2	[41]
GO-CTS	5	298	1000	50.51	[42]
PTFG-4	6	293	250	140.7	[43]
NH_3_-GO	6	298	1000	80.13	[44]
GO-COOH	5	298	50	344.1	This work

## Data Availability

The data can be made available upon reasonable request.
